# An effective content-based image retrieval technique for image visuals representation based on the bag-of-visual-words model

**DOI:** 10.1371/journal.pone.0194526

**Published:** 2018-04-25

**Authors:** Safia Jabeen, Zahid Mehmood, Toqeer Mahmood, Tanzila Saba, Amjad Rehman, Muhammad Tariq Mahmood

**Affiliations:** 1 Department of Software Engineering, University of Engineering and Technology, Taxila, Pakistan; 2 Department of Computer Science, University of Engineering and Technology, Taxila, Pakistan; 3 College of Computer and Information Sciences, Prince Sultan University, Riyadh, Saudi Arabia; 4 College of Computer and Information Systems, Al-Yamamah University, Riyadh, Saudi Arabia; 5 School of Computer Science and Engineering, Korea University of Technology and Education, Cheonan, Republic of Korea; Stanford University Medical Center, UNITED STATES

## Abstract

For the last three decades, content-based image retrieval (CBIR) has been an active research area, representing a viable solution for retrieving similar images from an image repository. In this article, we propose a novel CBIR technique based on the visual words fusion of speeded-up robust features (SURF) and fast retina keypoint (FREAK) feature descriptors. SURF is a sparse descriptor whereas FREAK is a dense descriptor. Moreover, SURF is a scale and rotation-invariant descriptor that performs better in the case of repeatability, distinctiveness, and robustness. It is robust to noise, detection errors, geometric, and photometric deformations. It also performs better at low illumination within an image as compared to the FREAK descriptor. In contrast, FREAK is a retina-inspired speedy descriptor that performs better for classification-based problems as compared to the SURF descriptor. Experimental results show that the proposed technique based on the visual words fusion of SURF-FREAK descriptors combines the features of both descriptors and resolves the aforementioned issues. The qualitative and quantitative analysis performed on three image collections, namely Corel-1000, Corel-1500, and Caltech-256, shows that proposed technique based on visual words fusion significantly improved the performance of the CBIR as compared to the feature fusion of both descriptors and state-of-the-art image retrieval techniques.

## Introduction

Due to the rapid growth of the internet and advancements in image acquisition devices, increasing amounts of visual data are created and stored, leading to an exponential increase in the volume of image collections. The techniques have been introduced to improve the effectiveness as well as efficiency of the content-based image retrieval (CBIR) systems [1–5]. CBIR is the mechanism by which a system retrieves images from an image collection according to the visual contents of the query image. These image retrieval techniques are based on either query by text or query by example. The image collections are difficult to mark semantically by textual labels due to advancements in digital cameras and social media which have resulted in an exponential increase in the size of the image collections. Furthermore, traditional annotation-based image retrieval techniques are language-dependent. To resolve such issues, researchers focus on retrieving images on the basis of the visual contents of the images. Low-level features like shape, texture, color, and spatial layout, and mid-level features like scale-invariant feature transform (SIFT), histograms of oriented gradients (HOG), etc. are used to retrieve images from an image collection. The challenges in the design of CBIR systems are bridging the spatial layout, overlapping objects, variations in illuminations, semantic gap, rotation and scale changes in images, and exponential growth of the image collections [6–11].

Researchers are currently concentrating on challenging problems in CBIR in different disciplines such as computer vision, pattern recognition, machine learning etc. The review on the most challenging issues in CBIR are presented in [12]. Some of the challenges in CBIR are the searching of objects from huge image repositories like ImageNet, ImageCLEF etc. There is absence of explicit phase of training to select features and to tune for classification. The semantic gap between visual contents and human semantics, the exponential growth in multimedia archives, the variation in illumination and spatial layout are some of the main reasons for making CBIR a challenging research problem [6, 7, 13].

**Fig 1:**
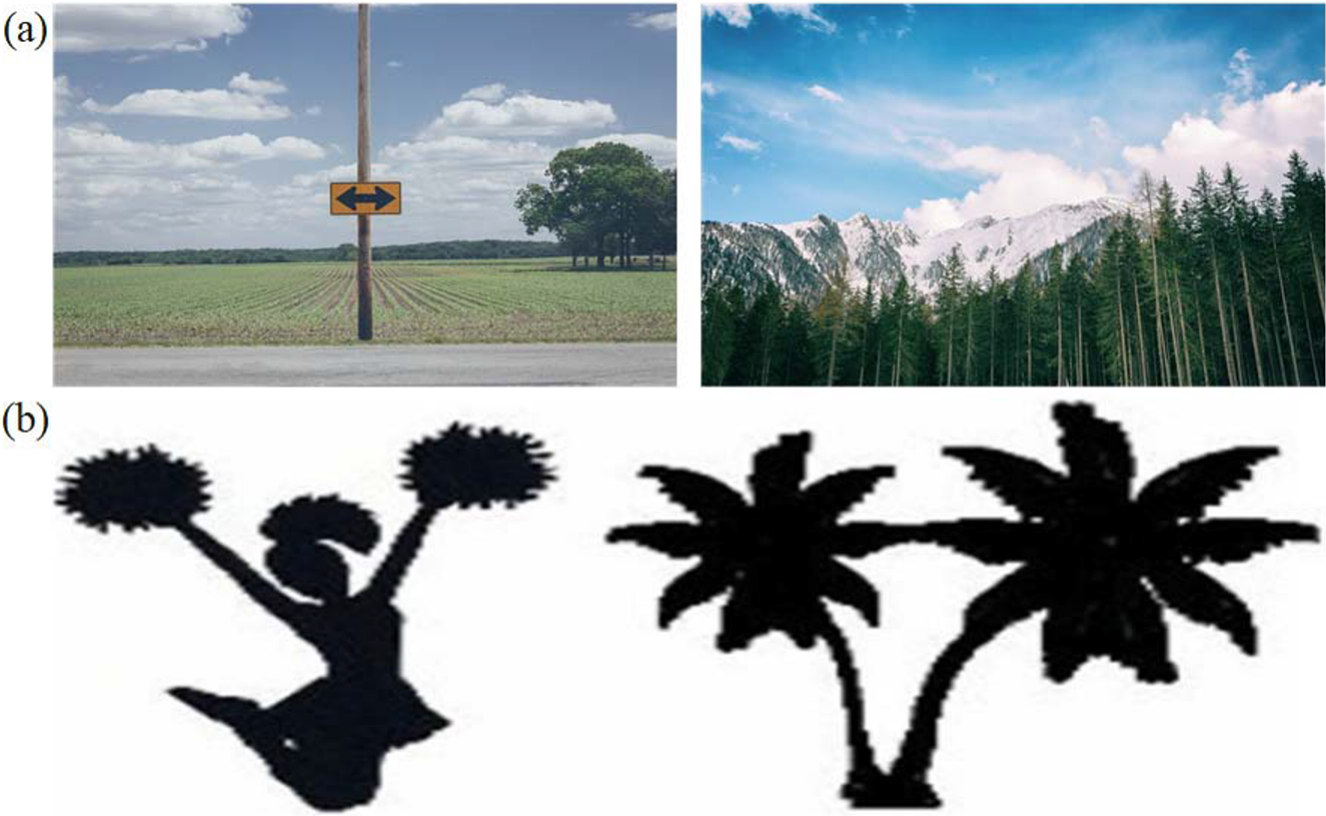
(a) Semantic gap-Corel images of two different semantic categories (i.e. “Mountains” and “Beach”) with close visual appearance; (b) Two sample images of different shapes with close visual and semantic appearance (images used in the figure are similar but not identical to the original images used in the study due to copyright issue, and is therefore for illustrative purposes only).

The difference which lies between high-level semantics and the low-level image features is known as the semantic gap [14]. The images shown in Fig 1(A) are taken from two different semantic categories of the Corel-1000 image collection. These images have a semantic likeness, close visual similarity, and matching colors which may increase the semantic gap, thus reducing the performance of the CBIR [14]. When a user enters a certain kind of query image, it is possible that the image on the left side (i.e. belonging to the semantic category of "Mountains") will be classified in the semantic category of “Beach” (e.g. sample image on the right) and vice versa, thus producing irrelevant results which also reduce the performance of the image retrieval. While low-level features, i.e. the colors of the visual contents of the images in Fig 1(A), are almost identical, the semantic information, i.e. beach or mountain, is not the same. Similarly, consider Fig 1(B) in which the palm tree looks roughly similar in shape to a cheerleader [14]. During the image matching process, the system can make wrong interpretations and might not provide what one actually wants in response to a user query due to the close visual appearance which reduces image retrieval performance. The main intention of retrieving images on the basis of the visual contents of the image is that they are in semantic correlation with the query image [6, 13, 15, 16].

The image representations based on the single type of local feature may produce unsatisfactory performance of CBIR due to inadequate representation of the visual contents of the images [17, 18]. In order to augment the effectiveness and reliability of image retrieval, different feature fusion or integration techniques have been introduced [17–20]. In this article, SURF and FREAK are considered as two strong local features. According to Bay et al. [21], SURF is a scale and rotation-invariant descriptor that performs better with respect to repeatability, distinctiveness, and robustness. It is also robust to noise, detection errors, geometric, and photometric deformations. Alahi et al. [22] state that FREAK is a fast, compact, and robust descriptor that performs well for classification-based problems and more suitable for the real-time image-matching applications [23].

The main objective of this paper is to present a novel technique based on visual words fusion or integration as well as the features fusion of SURF-FREAK feature descriptors on the basis of the bag-of-visual-words (BoVW) model in order to reduce the issue of the semantic gap and to improve the image retrieval performance. Firstly, the local features are computed from the sets of training and test images using SURF-FREAK feature descriptors. By applying *k-*means++ clustering algorithm [24] on each descriptor's extracted features, the high dimensional feature space of each descriptor is reduced to clusters, also known as visual words, to formulate the dictionary separately for SURF-FREAK feature descriptors. After that, visual words of both descriptors are fused together by integrating dictionaries of both feature descriptors. Then a histogram is constructed using the fused visual words of each image. The learning of the classifier is performed using histograms of training images. The similarity between the query image (which is taken from the test image set) and the images stored in an image collection is measured by applying Euclidean distance [20].

The following are the main contributions of this article:

1. Visual words fusion of SURF-FREAK feature descriptors based on the BoVW methodology.
2. Features fusion of SURF-FREAK feature descriptors based on the BoVW methodology.
3. Reduction of the semantic gap between low-level features of the image and high-level semantic concepts.

The remaining sections of this research article are organized as follows: a literature review of the state-of-the-art CBIR techniques is described concisely in Section-2. The detailed methodology of the proposed technique is discussed in Section-3. Section-4 presents experimental details and performance measurements of the proposed CBIR technique on three image collections followed by an analysis of the computational complexity. Finally, Section-5 concludes the proposed technique.

## Literature review

The main aim of CBIR is to search large repositories of images in order to retrieve images corresponding to the visual contents of the given query image. The accuracy and efficiency are the important attributes while retrieving the images on the basis of visual contents. Visual features such as global and local features are two basic categories on which conventional CBIR techniques are based [25–27].

Mathew et al. [28] propose an efficient CBIR technique which is based on shape signatures to retrieve relevant images from an image collection according to the semantic category of the query image. Abbadeni et al. [29] propose the well-known autoregressive model (AR) for texture features representation. They present the synthesis algorithm and estimated measurement of the degree of consistency of textures. Xu et al. [30] propose the manifold ranking (MR) model for image retrieval which is computationally expensive. It restricts its implementation to larger databases specifically to those cases in which the query image does not belong to the semantic category of the image collection. Subsequently, they proposed an innovative accessible graph-based ranking model known as efficient manifold ranking (EMR) which addresses the deficiencies of MR model from two main perceptions, namely that the construction of accessible graphs and efficient ranking of images during image retrieval process requires a high computational cost. Guo and Prasetyo [31] propose a technique for CBIR which involves manipulating the gain of ordered dither block truncation coding (ODBTC) method in order to produce an efficient feature representation of each image to improve the performance of the image retrieval. Liao et al. [32] propose a new variant of the SIFT descriptor. They regulate elliptical neighboring regions and use a polar histogram orientation bin. Moreover, they transform the affine scale space and integrate mirror reflection invariance in order to address the issue of the semantic gap of CBIR. Abdel-Hakim et al. [33] introduce a novel feature descriptor for CBIR known as colored SIFT (CSIFT) which has photometrical and color invariant features as compared to conventional SIFT feature descriptor which is particularly designed for grayscale images and which ignores the color characteristics of an image. Color and geometric information for object description are combined in the proposed descriptor for an effective CBIR. Mehmood et al. [15] propose a novel image representation technique for CBIR based on local and global histograms of each image. They divide the image into local rectangular regions to construct a local histogram while a global histogram is constructed using visual information of the whole image. Spatial information about salient objects within each image is retained in order to improve the performance of the image retrieval and overcome the semantic gap issue.

Zeng et al. [34] propose a novel image representation technique to represent an image in the form of a spatiogram. A Gaussian mixture model (GMM) is used to calculate the generalized histogram. Color space is computed by applying the expectation-maximization (EM) technique. The Bayesian information criterion (BIC) is used to compute the number of Gaussian components. Moreover, spatiogram illustration is incorporated with quantized GMM. Yuan et al. [17] propose a novel technique by integrating SIFT and local binary patterns (LBP) features based on the bag-of-features (BoF) model for image retrieval. The proposed framework outperformed on the images with noisy background and illumination changes. They propose path-based and image-based models. Tian et al. [35] propose an efficient image retrieval technique based on an edge orientation difference histogram (EODH) descriptor. EODH is a rotation and scale-invariant descriptor. The EODH features are integrated with color-SIFT features in order to achieve the effective performance of image retrieval. Yildizer et al. [36] propose a novel CBIR technique using a multiple support vector machines ensemble. The 2D Daubechies wavelet transformation is applied for the feature extraction process. Poursistani et al. [37] propose an efficient technique for image indexing and retrieval. The vector quantization based features are extracted from compressed JPEG images. The dictionary is formulated by applying *k*-means clustering technique. The proposed technique is able to construct a histogram from DCT coefficients which are considered the major component of JPEG compression.

## Proposed methodology

**Fig 2:**
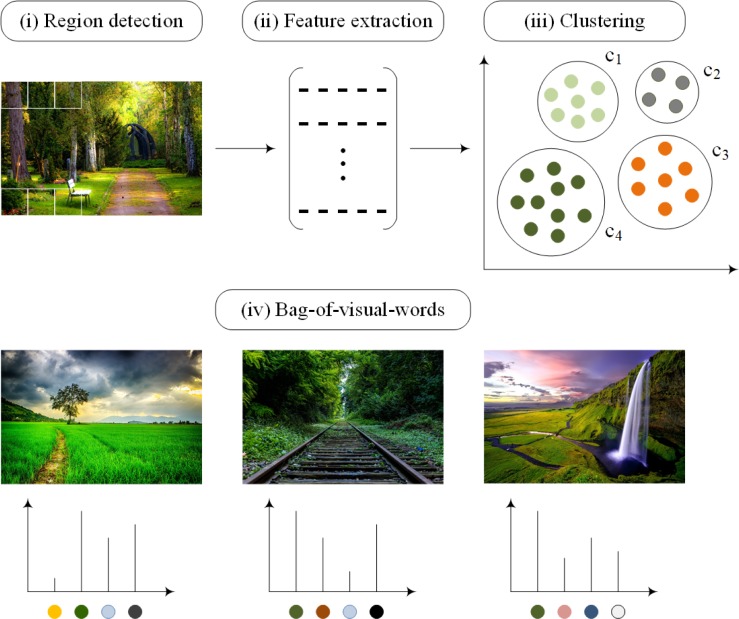
Methodology of the BoVW based image representation for CBIR.

**Fig 3:**
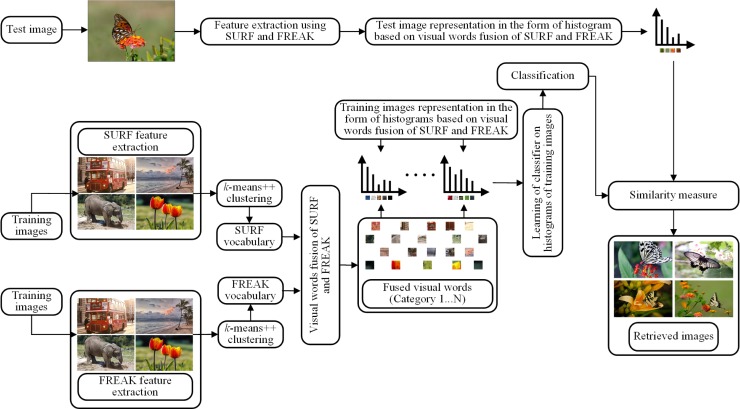
Block diagram of the proposed technique based on visual words fusion (images used in the figure are similar but not identical to the original images used in the study due to copyright issue, and is therefore for illustrative purposes only).

The foremost objective of the proposed CBIR technique based on visual words fusion is to decrease the semantic gap between high-level semantic and low-level image features and to improve the performance of the CBIR. The visual words fusion of SURF-FREAK feature descriptors is performed in order to achieve this goal. The methodology of the image representation based on the BoVW model is shown in Fig 2. The methodology of the proposed technique based on visual words fusion is presented in Fig 3.

The methodology of the proposed technique is described as follows:

1. Consider an image *h* that is composed of pixels and any pixel *p* at position (*i*,*j*) is represented by:
  $$h=(p_{i,j})$$ (1)
2. The images of each image collection are divided into training and test sets, SURF-FREAK features are extracted from these images. The Fast-Hessian matrix is formulated by applying a blob detector [21] for the detection of SURF keypoints. Consider a point *p* = (*i*,*j*) in an image *h*, the Hessian matrix *M*(*i*,*σ*) in *p* at scale *σ* is defined as follows:
  $$M(p,\sigma )=\left[\begin{array}{cc} L_{ii(p,\sigma )} & L_{ij(p,\sigma )}\\ L_{ij(p,\sigma )} & L_{jj(p,\sigma )} \end{array}\right]$$ (2)
  where *L*_*ii*(*p*,*σ*)_ is the convolution of the Gaussian second order derivative $h(i)\;\frac{\partial^{2}}{\partial i^{2}}g(\sigma )$ with the image *h* at point *p* and similarly for *L*_*ij*(*p*,*σ*)_, *L*_*ji*(*p*,*σ*)_, and *L*_*jj*(*p*,*σ*)_. These derivatives are known as the Laplacian of Gaussians. After detection of keypoints, SURF descriptors or features are computed at those extracted keypoints. Extracted features form the feature vector, which is represented as *FV*_*surf*_.
  $${FV}_{surf}=\{s_{1},s_{2},s_{3},\ldots .{\ldots s}_{n}\}$$ (3)
  where *s*_1_ to *s*_*n*_ are the computed SURF features. The dimensions of the SURF feature descriptor are 64 × *N*. Where *N* represents the number of extracted features, whose details are mentioned in Table 1 of the section 4.
  There are three main steps to compute FREAK features [22]: a sampling pattern, orientation compensation, and sampling pairs. For detecting the keypoints, the proposed method uses blob detector to formualte a Fast-Hessian matrix [21], which is represented by the following mathematical equation:
  $$M(i,j)=\left[\begin{array}{cc} h_{i}^{2}(i,j) & h_{i}h_{j}(i,j)\\ h_{i}h_{j}(i,j) & h_{j}^{2}(i,j) \end{array}\right]$$ (4)
  where *M* is the matrix that stores the keypoints, while *h*_*i*_ and *h*_*j*_ are the image derivatives in the *i* and *j* directions, respectively. The FREAK features or descriptors from an image *h* are computed and represented by the following mathematical equations:
  $$f_{x=}f(i_{x},j_{x})=L_{r_{x}}(i_{x},j_{x})$$ (5)
  $$where\;L_{r_{x}}(i,j)=h(i,j)*G_{r_{x}}(i,j,\sigma_{r_{1}})$$ (6)
  where *h*(*i*,*j*) is the input image, $G_{r_{x}}(i,j,\sigma_{r_{1}})$ is the Gaussian kernel for the *x*^th^ receptive field (where *x* = 1,2,3….*n*) and $L_{r_{x}}(i,j)$ represents the smoothed version of the input image. The *x*^th^ sampling point *f*_*x*_ corresponding to the center of the *x*^th^ receptive field *r*_*x*_ is defined using the predefined coordinates (*i*_*x*_,*j*_*x*_) from the sampling pattern. The computed features form the feature vector of FREAK, which is represented as *FV*_*freak*_ are mathematically defined by the following equation:
  $${FV}_{freak}=\{f_{1},f_{2},f_{3},\ldots .{\ldots f}_{n}\}$$ (7)
  where *f*_1_ to *f*_*n*_ are the computed FREAK features. The dimensions of the FREAK feature descriptor are 64 × *N*. Where *N* represents the number of extracted features, whose details are also mentioned in Table 1 of the section 4.
3. After extracting the features by applying SURF and FREAK descriptors on each image, the feature percentage from each descriptor is calculated by applying following mathematical equation:
  $$R_{fv}=D_{e}*R_{fp}$$ (8)
  where *D*_*e*_ represents the extracted feature of the descriptor, *R*_*fp*_ represents required feature percentage (0 < *R*_*fp*_ ≤ 1), and *R*_*fv*_ represents the resultant feature vector.
4. The *k*−means++ [24] clustering algorithm takes feature space as input and reduces it into clusters as output. The center of each cluster is called a visual word and the combination of visual words formulates the dictionary, which is also known as codebook or vocabulary. The dictionary that consists of clusters or visual words is formulated by applying the following mathematical equation on the extracted features:
  E(c1…..ck)=∑j=1E∑k=1Ch(xjϵCk)‖xj−ck‖^2 (9)
  where *C* is the subsets of clusters, *j* is the initial position of the cluster center, *C*_*k*_ is the number of clusters, the sum of the squares of the Euclidean distances between each data point *x*_*j*_ and centroid *c*_*k*_ is ‖*x*_*j*_ − *c*_*k*_‖^2. The clustering error *E* depends on cluster centers *c*_1_…..*c*_*k*_.
  The formulated dictionary of the SURF visual words is represented as follows:
  $$\left\{{FV}_{surf}\}\overset{k-means++}{\to }\{{VW}_{s}|s=1,2,3,\ldots ,c_{n}\right\}$$ (10)
  where *VW*_*s*_ is the set of computed visual words which forms a dictionary for the SURF descriptor that is represented as VW_*s*_ = *D*_*SURF*_ and *c*_*n*_ is the total number of visual words.
  The formulated dictionary of the FREAK visual words is represented as follows:
  $$\left\{{FV}_{freak}\}\overset{k-means++}{\to }\{{VW}_{f}|f=1,2,3,\ldots ,c_{m}\right\}$$ (11)
  where *VW*_*f*_ is the set of computed visual words which forms a dictionary for the FREAK descriptor that is represented as *VW*_*f*_ = *D*_*FREAK*_ and *c*_*m*_ is the total number of visual words.
  After that, the visual words of the both feature descriptors that are now in the form of two dictionaries (i.e. *D*_*SURF*_ and *D*_*FREAK*_) are vertically fused together and represented by the resultant dictionary *D*, which is represented by the following mathematical equation:
  $$D=\{D_{SURF};D_{FREAK}\}$$ (12)
5. For the proposed technique which is based on a simple feature fusion of SURF and FREAK descriptors, SURF and FREAK features are extracted separately from each image in the training and test sets, whose details are mentioned earlier in steps 1–2, respectively. After that, extracted features are vertically fused or concatenated together. Then the *k*-means++ [24] clustering technique is applied to the fused features, which formulate a single dictionary that consists of visual words. By using these visual words of the dictionary, the histogram is constructed using fused visual words of each image. The support vector machine (SVM) classifier is trained using histograms of training images and images are retrieved by applying the similarity measure technique between the score of the query image (which is taken from the test image set) and the images stored in an image collection.
6. The performance of the proposed technique is also analyzed using adaptive/weighted feature fusion (WFF) of the SURF-FREAK feature descriptors, which is mathematically represented as follows:
  $$WFF=\frac{w\times {FV}_{surf}+(1-w)\times {FV}_{freak}}{2}$$ (13)
  The values of the weight *w* are given in Table 2.
7. According to the experimental details given in Section 4, the proposed technique based on visual words fusion outperforms as compared to the adaptive feature fusion technique, simple feature fusion technique, and state-of-the-art CBIR techniques. Because the size of the dictionary in the case of visual words fusion is twice as large (i.e. two dictionaries are formulated), it represents the visual contents of the images in a more effective or compact form as compared to the size of the dictionary in the case of the feature fusion technique, in which a single dictionary is formulated.
8. The mapping of each visual word is done over the image by assigning the nearest visual words to the quantized descriptors using the following mathematical equation:
  $$VW(d_{k})={argmin\;Dist}_{VW\in D}(VW,d_{k})$$ (14)
  where *D* represent the resultant dictionary after the visual words fusion of both descriptors, *VW*(*d*_*k*_) represents the visual word assigned to the *k*^*th*^ descriptor *d*_*k*_, while *Dist*(*VW*,*d*_*k*_) is the distance between the descriptor *d*_*k*_ and the visual word *VW*.
9. The histogram of *v* visual words is formed from each image. After that, resultant information in the form of histograms is added to the inverted index of the BoVW model.
10. The histograms are normalized and the Hellinger kernel function (which is selected due to low computational complexity) of the SVM [38] is applied on the normalized histograms, represented by the following mathematical equation in order to train the SVM classifier:
  $$K(h,h^{\prime })=\sum_{i}\sqrt{h(i)h^{\prime }(i)}$$ (15)
  where in above equation, normalized histograms are represented by *h* and *h*′of the *i*^*th*^ image.
11. The similarity measure technique based on Euclidean distance is applied to retrieve relevant images according to the response of the given query image, which is represented mathematically by:
  D(Xm−Xn)=∑i=1N(Xim−Xin)^2 (16)
  where feature descriptors of the query image are represented by *X*^*m*^, and *X*^*n*^ represents the feature descriptors of the images stored in an image collection and *N* ∈ *FV*_*FREAK*_ and *FV*_*SURF*_.

## Experimental results and discussions

This section deals with the assessment of the technique presented on the basis of the experiments performed. All the images are processed in grayscale in order to save the computational complexity and experimental results are reported after repeating every experiment 10 times. For each experiment, images are grouped into training and testing sets. The dictionary is constructed using all of the images from the training set and performance is tested by taking images from the testing set of each image collection. In the proposed technique, dictionary size and features percentages per image are two important parameters which affect the performance of the CBIR. While the size of the dictionary is directly proportional to the performance of the CBIR, larger sizes of the dictionary produce the problem of overfitting in CBIR. In order to evaluate the best performance of the proposed technique, dictionaries of different sizes (i.e. 20, 50 100, 200, 400, 600, 800, 1000, 1200) are formulated using different features percentages (i.e. 10%, 25%, 50%, 75%, and 100%) per image.

### Parameters of the performance evaluation metrics

The performance of the proposed technique is evaluated using the precision, recall, and precision-recall (PR) curve parameters which are most widely used to evaluate the performance of the state-of-the-art CBIR techniques. The precision *P* measures the accuracy of the CBIR techniques, which is mathematically represented as follows:

$$P=\frac{C_{r}}{T_{r}}$$ (17)

where *C*_*r*_ represents the number of relevant images among retrieved images and *T*_*r*_ represents the total number of retrieved images.

The recall *R* measures the robustness of the CBIR techniques and is mathematically represented as follows:

$$R=\frac{M_{r}}{T_{p}}$$ (18)

where *M*_*r*_ represents number of relevant images among retrieved images and *T*_*p*_ represents the total number of images in a particular semantic category.

### Performance analysis on the Corel-1000 image collection

**Fig 4:**
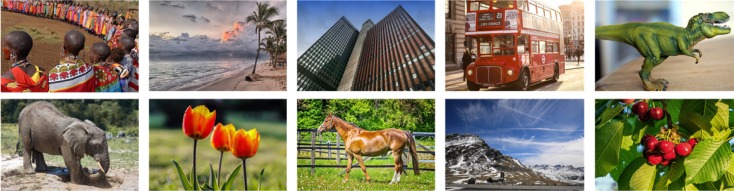
Sample of images from different semantic categories of the Corel-1000 and Corel-1500 image collections (images used in the figure are similar but not identical to the original images used in the study due to copyright issue, and is therefore for illustrative purposes only).

The performance of the proposed technique based on visual words fusion is estimated on the Corel-1000 image collection and the results are compared with state-of-the-art CBIR techniques [15, 20, 34, 39, 40] as well as with features fusion, standalone SURF, and standalone FREAK CBIR techniques based on the BoVW methodology. The Corel-1000 image collection [16, 41] is comprised of 1000 images and the resolution of each image is either 256 × 384 or 384 × 256. These images are grouped into 10 semantic categories. Every semantic category is composed of 100 images. Fig 4 represents a sample of images from different semantic categories of the Corel-1000 and Corel-1500 image collections.

For performance analysis on the Corel-1000 image collection, images are categorized into two groups known as the training and test sets which contain 70% and 30% of the images, respectively. Different sizes of the dictionary (i.e. 20, 50 100, 200, 400, 600, 800, 1000, and 1200) are built using images from a training set. Table 1 presents performance analysis in terms of the mean average precision (MAP) of the proposed technique based on visual words fusion on different sizes of the dictionary using different percentages of the features. According to the experimental details shown in Table 1, the best performance in terms of average precision (AP) is 86%, which is achieved on a dictionary size of 800 visual words using 50% features per image. The statistical analysis is performed using Wilcoxon matched-pairs signed-rank test that presents the robustness of statistical results in terms of *P* and *Z* values and the value of *P* is less than the value of significance level (i.e. ∝ ≤ 0.05) on all the reported dictionary sizes. The statistical results are reported by comparing the performance on a dictionary size of 800 visual words with other reported dictionary sizes (i.e. 20, 50, 100, 200, 400, 600, 1000, and 1200) as well as with [34] for a dictionary size of 800 visual words.

**Table 1:** Performance comparison and statistical analysis of different sizes of the dictionary and features percentages of the image on the Corel-1000 image collection (bold values indicate best performance).

Features % per image	Performance analysis in terms of the MAP performance (in %) on the different sizes of the dictionary								
	20	50	100	200	400	600	800	1000	1200
**10%**	74.06	75.60	79.53	79.86	80.76	80.76	80.86	80.85	80.53
**25%**	76.46	78.33	81.16	80.93	81.16	80.99	81.26	81.19	81.25
**50%**	73.93	78.19	80.60	81.19	81.93	82.30	**86.00**	80.66	81.13
**75%**	74.40	77.93	80.40	82.05	81.73	82.05	82.06	81.00	81.33
**100%**	75.60	76.80	80.26	82.40	80.73	82.20	82.40	81.13	81.53
**MAP**	74.89	77.37	80.39	81.28	81.26	81.66	**82.51**	80.96	81.15
**Std. dev.**	1.097	1.158	0.590	0.999	0.550	0.726	2.042	0.215	0.377
**Confidence interval**	73.52- 76.25	75.93- 78.80	79.65- 81.12	80.04- 82.52	80.57- 81.94	80.75- 82.56	80.80- 83.10	80.69- 81.23	80.68- 81.62
**Std. error**	0.490	0.517	0.263	0.446	0.246	0.324	0.413	0.096	0.169
**Non-parametric statistical analysis using Wilcoxon matched-pairs signed-rank test**									
**Z-value**	2.023	2.023	2.023	2.023	2.023	2.023	2.023	2.023	2.023
**P-value**	0.043	0.043	0.043	0.043	0.043	0.043	0.043	0.043	0.043

**Fig 5:**
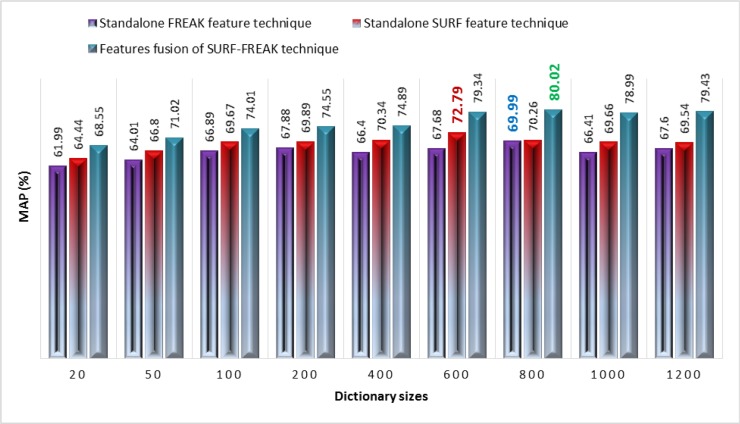
Performance comparisons of standalone SURF, standalone FREAK, and features fusion of SURF and FREAK descriptors techniques on different sizes of the dictionary for the Corel-1000 image collection.

The performance analysis in terms of MAP of standalone SURF, standalone FREAK, and features fusion of SURF-FREK descriptors techniques based on the BoVW methodology is presented in Fig 5. According to the experimental results shown in Fig 5, the proposed technique based on features fusion of SURF-FREAK performs better on all of the reported dictionary sizes due to the fusion of both descriptors features as compared to the performance of the standalone SURF feature and standalone FREAK feature techniques based on the BoVW methodology.

**Fig 6:**
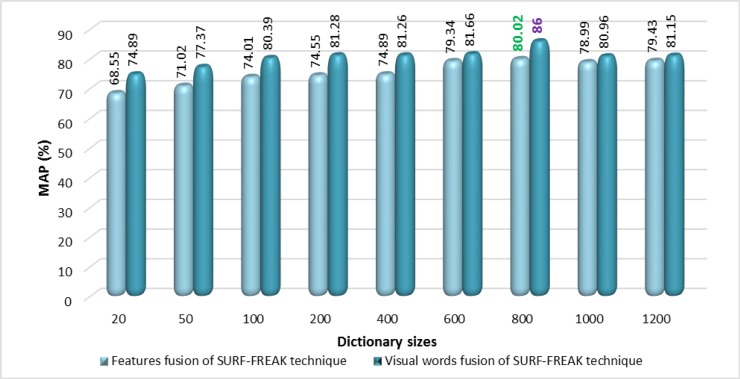
Comparison between visual words fusion vs. features fusion of SURF-FREAK using proposed technique on the Corel-1000 image collection.

The best values of the performance in terms of MAP for the proposed technique based on visual words fusion, features fusion, standalone SURF, and standalone FREAK techniques are highlighted in Figs 5 and 6 for the Corel-1000 image collection. The experimental results of the proposed technique based on visual words fusion are reported in Fig 6, which are compared with the proposed technique based on features fusion of SURF-FREAK as well as with state-of-the-art CBIR techniques [15, 20, 34, 39, 40]. The proposed technique based on visual words fusion performs better because the size of the dictionary is twice as large (due to the formulation of two dictionaries to present fused visual words), as it represents image features in the form of visual words in a more compact form as compared to the features fusion technique which represents fused SURF-FREAK descriptor features by formulating a single dictionary.

The experimental details of the proposed technique based on the adaptive feature fusion on different sizes of the dictionary are given in Table 2. The best MAP performance of the adaptive feature fusion technique is achieved on a dictionary size of 1000 visual words, which is 74.05%.

**Table 2:** Performance analysis using adaptive or weighted feature fusion of SURF-FREAK descriptors on the Corel-1000 image collection (bold values indicate best performance).

Weight *W* of SURF-FREAK	MAP performance (in %) on the different sizes of the dictionary								
	20	50	100	200	400	600	800	1000	1200
1.0–0.0	64.44	66.80	69.67	69.89	70.34	72.79	70.26	69.66	69.54
0.9–0.1	65.03	66.91	70.16	70.31	70.82	72.89	72.16	72.81	69.21
0.8–0.2	65.14	67.11	70.23	70.43	70.91	72.99	72.81	72.99	68.02
0.7–0.3	65.23	67.24	70.29	70.63	71.23	73.11	73.02	73.39	67.91
0.6–0.4	65.45	67.38	70.38	70.71	71.41	73.23	73.59	73.82	67.73
0.5–0.5	65.51	67.48	70.48	70.84	71.63	73.51	73.81	73.98	67.71
**0.4–0.6**	65.59	67.88	70.83	71.03	71.89	73.83	73.89	**74.05**	67.68
0.3–0.7	65.43	67.83	70.81	70.98	71.83	73.78	73.51	74.01	67.66
0.2–0.8	65.39	67.76	70.62	70.91	71.65	73.68	73.32	73.93	67.64
0.1–0.9	64.21	67.09	70.06	70.86	71.51	73.53	72.08	73.82	67.62
0.0–1.0	61.99	64.01	66.89	67.88	66.40	67.68	69.99	66.41	67.60

The training set for each image collection is formulated by taking the percentage of the images as shown in Table 3 from each semantic category of the reported image collections while remaining images from each semantic category are used for the formation of the test set for each reported image collections.

**Table 3:** Experimental details of the reported image collections for the proposed technique.

Name of the image collection	Total semantic categories	Total images within the collection	% of training set of the images	% of test set of the images
**Corel-1000**	10	1000	70%	30%
**Corel-1500**	15	1500	50%	50%
**Caltech-256**	256	30,607	60%	40%

**Fig 7:**
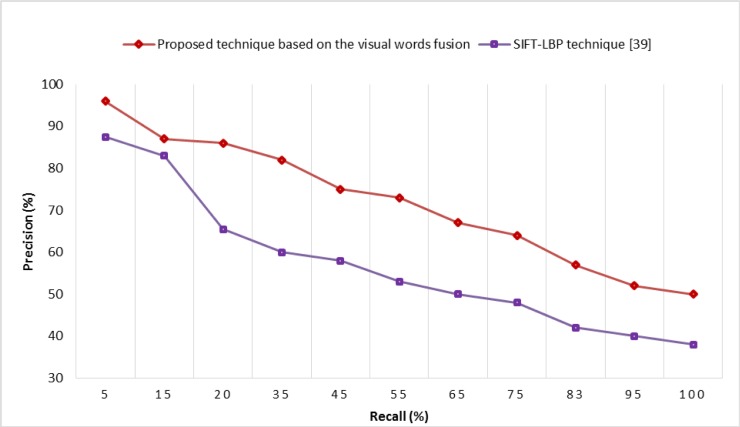
Performance comparison in terms of PR-curve on the Corel-1000 image collection.

Tables 4 and 5 present a semantic category-wise comparative analysis in terms of MAP and average recall of the proposed technique based on visual words fusion (on a dictionary size of 800 visual words, using 100% features per image) with state-of-the-art CBIR techniques. In order to prove the robustness of the proposed technique based on visual words fusion of SURF-FREAK, the comparative analysis of performance in terms of PR-curve is performed with SIFT-LBP technique [42] on the Corel-1000 image collection, whose experimental details are shown in Fig 7. Fig 7 clearly indicates that the proposed technique based on visual words fusion of SURF-FREAK yields better performance as compared to the state-of-the-art CBIR technique [42].

**Table 4:** Semantic category-wise comparative analysis of proposed technique based on visual words fusion with state-of-the-art CBIR techniques by formulating dictionary size of 800 visual words on the Corel-1000 image collection (bold values indicate category-wise best performance).

Semantic categories	Zeng et al. [34]	Douik et al. [20]	Feng et al. [40]	ElAlami et al. [39]	Mehmood et al. [15]	Proposed technique
**Africa**	72.50	76.00	**87.50**	72.60	73.03	82.88
**Beach**	65.20	45.00	68.33	59.30	**74.58**	67.88
**Buildings**	70.60	59.00	61.67	58.70	80.24	**80.64**
**Buses**	89.20	**98.00**	80.00	89.10	95.84	97.48
**Dinosaurs**	**100.0**	**100.0**	**100.0**	99.30	97.95	98.61
**Elephants**	70.50	64.00	67.50	70.20	**87.64**	70.01
**Flowers**	94.80	96.00	88.33	92.80	85.13	**100.0**
**Horses**	91.80	93.00	**100.00**	85.60	86.29	97.44
**Mountains**	72.25	59.00	55.00	56.20	**82.43**	71.06
**Food**	78.80	61.00	74.17	77.20	78.96	**94.00**
**MAP**	80.57	75.00	78.25	76.10	84.21	**86.00**

**Table 5:** Semantic category-wise comparative analysis of recall with state-of-the-art CBIR techniques on the Corel-1000 image collection (bold values indicate category-wise best performance).

Semantic categories	Zeng et al. [34]	Douik et al. [20]	Feng et al. [40]	ElAlami et al. [39]	Mehmood et al. [15]	Proposed technique
**Africa**	14.50	15.20	10.50	16.10	14.61	**16.57**
**Beach**	13.04	09.00	08.20	**19.30**	14.92	13.57
**Buildings**	14.12	11.80	07.40	**19.10**	16.05	16.13
**Buses**	17.84	**19.60**	09.60	12.60	19.17	19.49
**Dinosaurs**	**20.00**	**20.00**	12.00	10.90	19.59	19.72
**Elephants**	14.10	12.80	08.10	16.30	**17.53**	14.00
**Flowers**	18.96	19.20	10.60	12.90	17.03	**20.00**
**Horses**	18.36	18.60	12.00	14.40	17.26	**19.48**
**Mountains**	14.45	11.80	06.60	**18.60**	16.49	14.21
**Food**	15.76	12.20	08.90	14.80	15.79	**18.80**
**Avg. Recall**	16.11	15.00	9.39	16.10	16.84	**17.19**

**Fig 8:**
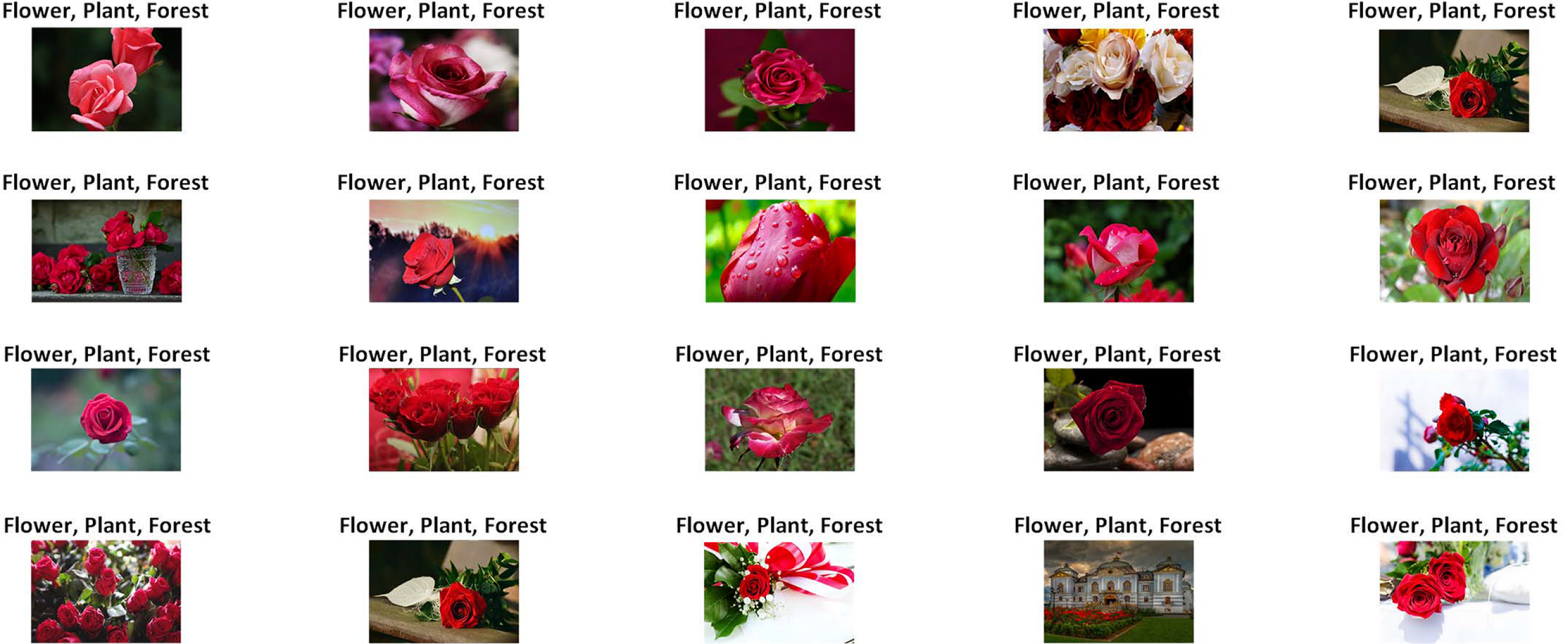
Image retrieval result shows a reduction of the semantic gap using automatic image annotation on the semantic category “Flowers” of the Corel-1000 image collection (images used in the figure are similar but not identical to the original images used in the study due to copyright issue, and is therefore for illustrative purposes only).

**Fig 9:**
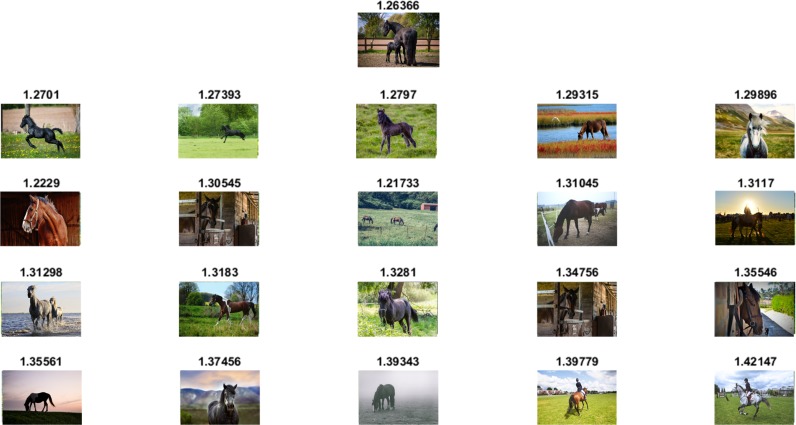
Image retrieval result shows a reduction of the semantic gap in the semantic category “Horses” of the Corel-1000 image collection (images used in the figure are similar but not identical to the original images used in the study due to copyright issue, and is therefore for illustrative purposes only).

The image retrieval results of the proposed technique based on visual words fusion for the semantic categories “Flowers” and “Horses” of the Corel-1000 image collection are shown in Figs 8 and 9. The numeric value shown at the top of each image is the score of the respective image. The image shown at the top of each Fig is the query image, while rest of the images are the retrieved images that are obtained by applying the Euclidean distance formula between a score of the query image and scores of the retrieved images. The images whose numeric values are more close to the score of the query image are more identical to the query image, which shows a reduction of the semantic gap between low-level features of the image and high-level image semantic concepts and vice versa. For showing reduction of semantic gap based on the automatic image annotation (AIA), the pre-defined semantic class annotations/labels that are utilized for the 10 classes of the Corel-1000 image benchmark are: “Restaurants”, “Food”, “Landscape”, “Mountains”, “Grass”, “Horses”, “Garden”, “Flowers”, “Forest”, “Elephants”, “Animals”, “Dinosaurs”, “Transport”, “Buses”, “Architecture”, “Buildings”, “Sky”, “Beach”, “People”, and “Africa”. The proposed technique perform AIA by assigning 3 annotations per image. Top classification score is obtained for each image and three pre-defined annotations are assigned on the basis of top classification score for each image according to the semantic class of the image as shown in Fig 8.

### Performance analysis on the Corel-1500 image collection

The Corel-1500 image collection [43] is comprised of 1500 images and the resolution of each image is either 256 × 384 or 384 × 256. These images are distributed into 15 semantic categories. Each semantic category contains 100 images.

**Fig 10:**
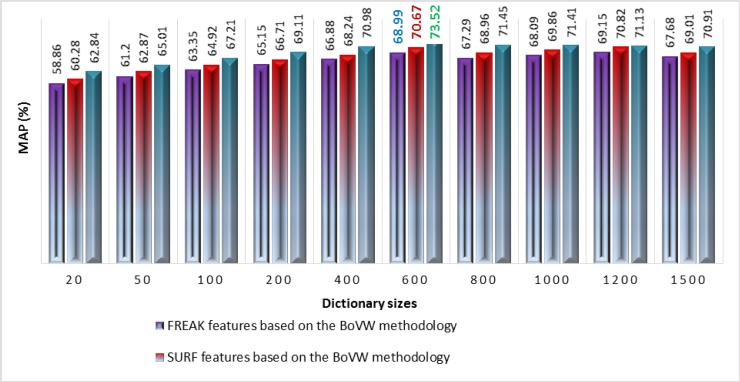
Performance comparisons of standalone SURF, standalone FREAK, and features fusion of SURF-FREAK techniques on different sizes of the dictionary for the Corel-1500 image collection.

**Fig 11:**
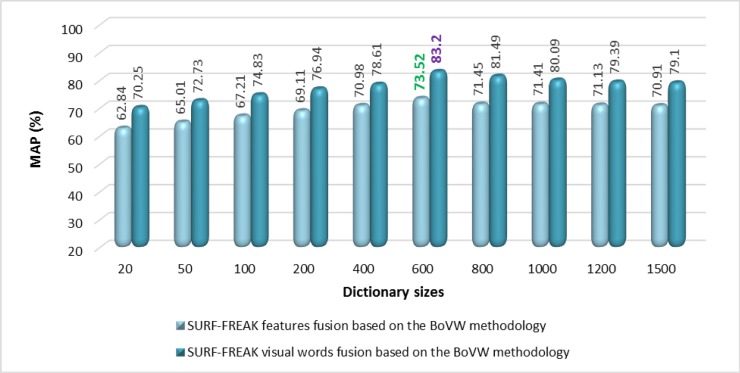
MAP performance comparison of the proposed technique based on visual words fusion vs. features fusion of SURF-FREAK technique on different sizes of the dictionary for the Corel-1500 image collection.

**Fig 12:**
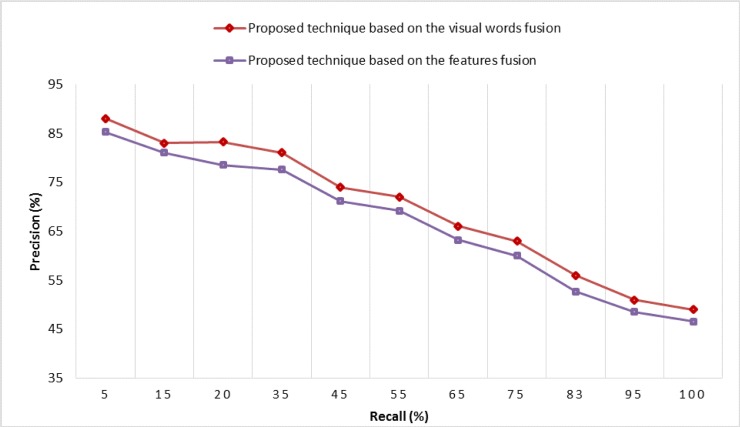
Performance comparison in terms of PR-curve on the Corel-1500 image collection.

Different sizes of the dictionary (i.e. 20, 50, 100, 200, 400, 600, 800, 1000, 1200, and 1500) are constructed using images of the training set. Fig 10 shows the performance analysis in terms of MAP for the standalone SURF features, standalone FREAK features, and features fusion of SURF-FREAK techniques based on the BoVW methodology on the Corel 1500 image collection. Fig 11 presents the performance analysis in terms of the MAP comparison between the proposed techniques based on visual words fusion of SURF-FREAK descriptors vs. features fusion of SURF-FREAK descriptors on the different reported sizes of the dictionary on the Corel 1500 image collection. According to the experimental results shown in Figs 10 and 11, and Table 6, the proposed technique based on visual words fusion outperforms as compared to the proposed technique based on the features fusion of the SURF-FREAK descriptors on a dictionary of all reported sizes as well as state-of-the-art CBIR technique [34]. The best MAP of 83.20% is achieved with a dictionary size of 600 visual words and using 50% of the features per image according to the experimental details of the proposed technique based on visual words fusion as shown in Figs 11 and 12 and Table 6.

**Table 6:** MAP and avg. recall comparisons with state-of-the-art CBIR techniques on the Corel-1500 image collection (bold values indicate best performance).

Performance measures	Proposed technique based on visual words fusion	GMM+mSpatiogram [34]	SQ+Spatiogram [34]
**MAP**	**83.20**	74.10	63.95
**Avg. Recall**	**16.64**	13.80	12.79

**Fig 13:**
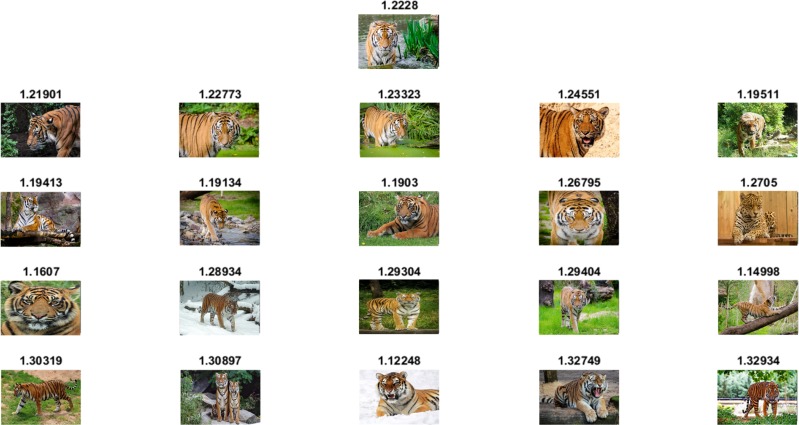
The retrieved images show a reduction of the semantic gap in response to the query image taken from the semantic category “Tigers” of the Corel-1500 image collection (images used in the figure are similar but not identical to the original images used in the study due to copyright issue, and is therefore for illustrative purposes only).

The top 20 image retrieval result of the proposed technique based on visual words fusion of SURF-FREAK descriptors is shown in Fig 13 for the semantic category “Tigers” of the Corel-1500 image collection.

### Performance analysis on the Caltech-256 image collection

The Caltech-256 image collection [44] constitutes 29,780 images and the resolution of each image is either 260 × 300 or 300 × 260. All of the images are in JPEG format. These images are categorized into 256 semantic categories like “Butterfly”, “Leopard”, “Hibiscus”, “Airplanes”, “Kitchen”, “Motorbike”, “Fireworks”, etc. Each semantic category contains 80 images.

**Fig 14:**
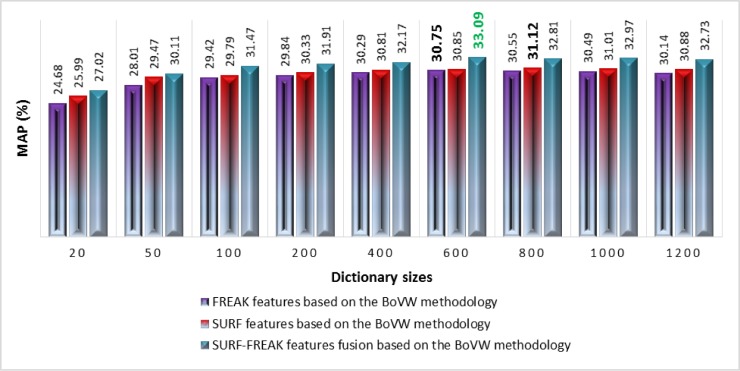
Performance comparisons of standalone SURF, standalone FREAK, and features fusion of SURF-FREAK techniques on different sizes of the dictionary for the Caltech-256 image collection.

For the Caltech-256 image collection, different sizes of the dictionary (i.e. 20, 50 100, 200, 400, 600, 800, 1000, and 1200) are constructed using images from a training set. Fig 14 shows the performance analysis in terms of MAP of the standalone SURF, standalone FREAK, and features fusion of SURF-FREAK feature descriptors techniques based on the BoVW methodology.

**Fig 15:**
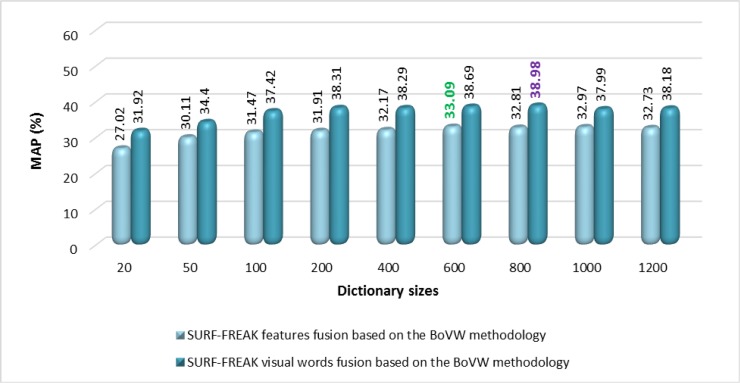
MAP performance comparison of the proposed technique based on visual words fusion vs. features fusion of SURF-FREAK techniques on different sizes of the dictionary for the Caltech-256 image collection.

**Fig 16:**
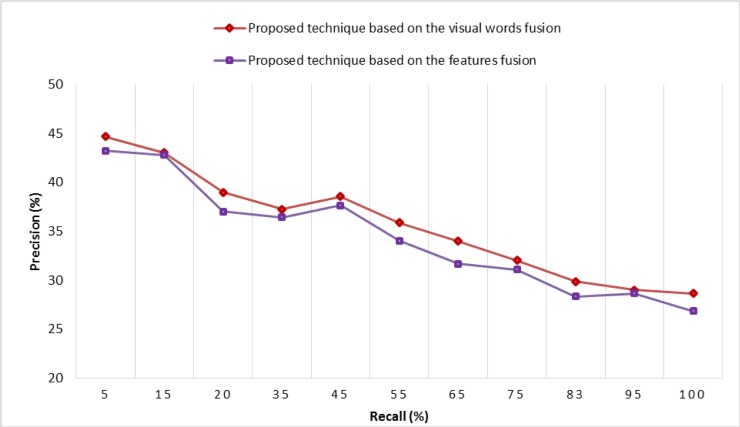
Performance comparison in terms of PR-curve on the Caltech-256 image collection.

According to the experimental details shown in Fig 14, the features fusion of SURF-FREAK technique outperforms on a dictionary of all the reported sizes as compared to the MAP performance of the standalone SURF and standalone FREAK techniques. Fig 15 renders a graphical representation of a performance comparison in terms of MAP between the proposed technique based on visual words fusion vs. the features fusion of SURF-FREAK feature descriptors technique on the Caltech-256 image collection. Fig 15 indicates that the proposed technique based on visual words fusion of SURF-FREAK feature descriptors gives a better performance than the feature fusion of SURF-FREAK technique on a dictionary of all the reported sizes. In order to prove the robustness of the proposed technique based on visual words fusion of SURF-FREAK, the comparative analysis of performance in terms of PR-curve is performed with features fusion of the SURF-FREAK technique on the Caltech-256 image collections, whose experimental details are shown in Fig 16.

For bestowing a sustainable performance of the proposed technique based on visual words fusion, the image retrieval performance measures (i.e. precision and recall) are also compared with the state-of-the-art CBIR techniques [45, 46]. Table 7 characterizes performance comparisons in terms of MAP and recall attained using the Caltech-256 image collection (on a dictionary size of 800 visual words and using 75% of the features per image) with the state-of-the-art techniques [45, 46] of CBIR.

**Table 7:** Performance measures comparisons with state-of-the-art CBIR techniques on the Caltech-256 image collection (bold values indicate best performance).

Performance measures	Proposed technique based on visual words fusion	Q-Way [45]	Dense SIFT [46]
**MAP**	**38.98**	38.56	38.57
**Avg. Recall**	**7.796**	7.712	7.714

The experimental results obtained on the Caltech-256 image collection prove the robustness of the proposed technique based on visual words fusion. According to the experimental details shown in Table 7, the proposed technique based on visual words fusion (on a dictionary size of 800 visual words and using 75% of the features per image) significantly outperforms as compared with the performance measures of the state-of-the-art CBIR techniques [45, 46].

### Performance analysis in terms of the computational complexity and required resources

This section presents performance analysis in terms of the computational complexity, required hardware and software resources for the proposed technique based on visual words fusion. The computational complexity is reported on a computer with the following hardware specifications: 4 GB RAM, Intel (R) Core (TM) i3-2310M 2.1 GHz CPU, Windows 7 64 bit operating system, solid-state drive (SSD) of capacity 120 GB. The algorithm of the proposed technique is implemented in MATLAB 2015a using VLFeat library (version 0.9.20). Table 8 presents the computational complexity (time in seconds) required for features extraction of the proposed technique based on the visual words fusion that is also compared with state-of-the-art CBIR techniques [35, 47] which also proofs the robustness of the proposed technique in terms of the computational complexity.

**Table 8:** Analysis of the computational complexity (time in seconds) required for feature extraction.

Proposed technique based on visual words fusion	Dubey et al. [47]			Tian et al. [35]
	RSHD	CDH	SHE	
**0.173**	0.375	1.709	0.186	5.6

The performance analysis of the proposed technique in terms of average required time for query image retrieval and its comparison with state-of-the-art CBIR techniques is presented in Table 9.

**Table 9:** Analysis of the computational complexity (time in seconds) required for query image retrieval (complete framework).

Retrieved images	Proposed technique based on the visual words fusion of SURF-FREAK	FBWN technique [48]	LGH technique [15]
Top 20 image retrieval	**0.7523**	0.87	0.7837

## Conclusion and future directions

In this article, we have proposed three novel techniques, known as visual words fusions, adaptive feature fusion, and simple feature fusion of SURF-FREAK feature descriptors based on the BoVW methodology in order to reduce the semantic gap between low-level features and high-level semantic concepts that effect CBIR performance. We can conclude that the proposed technique based on visual words fusion significantly improves the performance of the CBIR as compared to the proposed technique based on adaptive and simple features fusion of SURF-FREAK, standalone SURF, and standalone FREAK techniques. The performance of CBIR is improved because the size of the dictionary in the case of visual words fusion technique is twice as large compared to features fusion, standalone SURF, and standalone FREAK techniques that formulate a single dictionary to represent visual contents of the image, containing features of the single descriptor. Furthermore, the resultant fused descriptor contains features of both descriptors in terms of fused visual words. In order to reduce the computational cost that is raised due to visual words fusion and features fusion of SURF-FREAK descriptors, we have proposed different feature percentages per image. In future, we plan to evaluate the performance of the proposed technique by incorporating spatial information and using deep learning.
